# O.4.4-9 Organized sport and physical activity for older adults – salutogenic settings-based recommendations

**DOI:** 10.1093/eurpub/ckad133.206

**Published:** 2023-09-11

**Authors:** Helena Ericson, Susanna Geidne

**Affiliations:** Örebro University; helena.ericson@oru.se; Örebro University; helena.ericson@oru.se

## Abstract

Sport and physical activity is important to address healthy aging. There are recommendations on how much physical activity people should do, but no recommendations for how organizations should organize activities to suit as many as possible.

The purpose of this study was to explore the characteristics of sport and physical activity initiatives that older adults participate in. Different ongoing sport and physical activity initiatives that involve older adults were investigated regarding their focus, organization, intensity and organizer, and in relation to their costs, booking opportunities and recruitment.

The study was conducted with a cross-sectional design using the Salutogenic Physical Activity Health Resources Questionnaire (SPAHRQ). The study included 27 different initiatives with 372 participants (60% women) ranging from 60 to 96 years of age. A health-promoting, salutogenic, settings-based approach, and specifically the concepts drop-in, drop-through and drop-over (Geidne and Quennerstedt, 2021) were used in discussing recommendations for the organization of sport and physical activity for older adults.

The main findings were that who (sports clubs or senior associations) organizes the sport and physical activity initiative seems to affect the characteristics of how (for example intensity and characteristics of the activities) it is organized and what characterizes the participants in it. Despite the differing characters of sport and physical activity initiatives, the majority of older adults are recruited by internal contacts like friends and family. The lowest costs are found in senior associations, leisure-focused initiatives, individual initiatives, and low-intensity activities.

In senior associations, most activities were booked per semester 60%. In sport clubs the most common booking system was per occasion, 40%.

In team sports (almost always in sport clubs), the most common way of booking was per semester (46%). Which older adults participate in which initiatives is explained mostly by the age and gender of the participants.

In conclusion, to attract as many older adults as possible, organizations should work with lowering the thresholds into, as well as within and between, organizations, and raise the threshold for dropping out of sports and physical activity.

**Support/Funding Source:**

The study was funded in a PhD-project at Örebro University.

